# CO-DESIGNING A STAGE 1B FEASIBILITY STUDY OF INPATIENT PALLIATIVE COMMUNITY HEALTH WORKER SUPPORT

**DOI:** 10.1093/geroni/igad104.1124

**Published:** 2023-12-21

**Authors:** Jung Kwak, Elizabeth Kvale, Sarah Mills, Snehal Patel

**Affiliations:** University of Texas at Austin, Austin, Texas, United States; Baylor College of Medicine, Houston, Texas, United States; UT Austin Dell Medical School, Austin, Texas, United States; UT Austin Dell Medical School, Austin, Texas, United States

## Abstract

Persons living with dementia (PLWD) are 2-3 times more likely than their non-dementia peers to be hospitalized annually and experience burdensome care transitions and treatments at the late stage of dementia. Minoritized PLWDs are significantly more likely to be hospitalized and experience frequent transitions between care settings, and also less likely to access palliative care. There is a pressing need to develop better models of care transition support for PLWDs and their family caregivers. Proactive palliative care can reduce unnecessary hospitalization and improve outcomes for PLWDs and family caregivers. Community health workers (CHW) can effectively bridge cultural gaps between vulnerable patients/families and formal care systems, and provide care navigation and coaching support. We are currently conducting a single-site randomized controlled pilot trial study to co-design with key stakeholders (Stage 1a), implement and assess (Stage 1b) the acceptability and feasibility of a palliative CHW support for hospitalized PLWDs and caregivers. To date, we have conducted a needs assessment with 34 PLWD/caregiver dyads, key informant interviews with 4 palliative care providers and 2 CHW and social care experts, and multiple stakeholder meetings with hospital frontline providers and CHW groups to develop intervention components and research implementation plans that address language and cultural barriers and healthcare provider and system level barriers experienced by PLWD and caregivers from minoritized and financially vulnerable backgrounds. In this presentation, we will describe stakeholder engagement and co-design process outcomes and preliminary findings on intervention fidelity, participant recruitment and retention, and PLWD/caregiver outcomes.

